# 凝胶中荧光颗粒原位电泳洗脱过量异硫氰酸荧光素用于图像分析

**DOI:** 10.3724/SP.J.1123.2022.04023

**Published:** 2022-07-08

**Authors:** Guohong CHEN, Zehua GUO, Yiren CAO, Liuyin FAN, Weiwen LIU, Yixin MA, Chengxi CAO, Qiang ZHANG

**Affiliations:** 1.上海交通大学电子信息与电气工程学院, 上海 200240; 1. School of Electronic Information & Electrical Engineering, Shanghai Jiao Tong University, Shanghai 200240, China; 2.上海交通大学附属上海第六人民医院, 上海 200240; 2. Shanghai 6th People’s Hospital, Shanghai Jiao Tong University, Shanghai 200240, China; 3.上海交通大学学生创新中心, 上海 200240; 3. Student Innovation Center, Shanghai Jiao Tong University, Shanghai 200240, China

**Keywords:** 电泳, 电洗脱, 荧光颗粒, 图像检测, electrophoresis, electrophoretic elution, fluorescent particles, image detection

## Abstract

去除荧光标记后残余荧光染料可以提高荧光颗粒检测的灵敏度、准确度和效率。该文发展了一种原位电泳洗脱(electrophoretic elution, EE)模型,用于在荧光标记后快速去除多余的荧光探针,实现荧光颗粒的灵敏检测。将牛血清蛋白(BSA)和磁珠(MBs)作为模式蛋白和微颗粒,混合孵育获得MBs-BSA,用异硫氰酸荧光素(FITC)对MBs-BSA标记,得到MBs-BSA_FITC_复合物。将含有多余FITC的MBs-BSA_FITC_溶液与低凝聚温度琼脂糖凝胶溶液按1:5的体积比混合,并将混合物凝胶和纯琼脂糖凝胶分段填充到电泳通道中。电泳过程中,利用颗粒尺寸与凝胶孔径的差异来保留MBs-BSA_FITC_,同时将游离的FITC洗脱。经过30 min的电泳洗脱,通道内多余的FITC清除率达到97.6%,同时目标颗粒荧光信号保留了27.8%。成像系统曝光时间为1.35 s时,电泳洗脱将颗粒与背景的荧光信号比(P/B ratio, PBr)从1.08增加到12.2。CCD相机的曝光时间增加到2.35 s,可以将PBr提高到15.5,可进一步实现对微弱荧光亮点的高灵敏检测。该模型有以下优点:(1)能对颗粒表面非特异性吸附的FITC实现有效洗脱,提高了检测的特异性;(2)能够将97%以上的游离FITC清除;(3) 30 min内能够使凝胶内的背景荧光大幅降低,提高了PBr和检测灵敏度。因此,该方法具有在凝胶中进行基于磁珠/荧光颗粒点的免疫检测、在免疫电泳或凝胶电泳中对蛋白质/核酸条带进行荧光染色等领域的应用潜力。

## Abstract (en)

The sensitivity, accuracy, and efficiency of fluorescent particle detection can be improved by purifying the fluorescent-dye-labeled particles. In this study, an in-site model of electrophoretic elution (EE) was developed for the facile and efficient removal of unconjugated fluorescent dyes after labeling reactions, thereby facilitating the sensitive fluorescent imaging of proteins captured by microbeads. First, bovine serum albumin (BSA) and magnetic beads (MBs) were chosen as the model protein and particles, respectively, and an MBs-BSA complex was synthesized by mixing the beads with the BSA solution. Second, excessive fluorescein isothiocyanate (FITC) was added to the EP tube with MBs-BSA suspension for the fluorescent labeling of BSA, and a labeled compound was obtained after 8-h incubation in the dark at 4 ℃. The unpurified MBs-BSA_FITC_ was obtained by removing the supernatant, leaving 5 μL of the residual solution in the EP tube. The obtained MBs-BSA_FITC_ solution was added to a 50-μL phosphate buffer solution (PBST, containing 0.01% Triton X-100, pH 7.4). Third, gel suspension was prepared by mixing the MBs-BSA_FITC_ solution with the low-gelling-temperature agarose gel (10 g/L) and filled into an electrophoresis channel. To demonstrate the high efficiency of the in-site model of EE for removing excessive FITC, a 10-mm hydrogel segment was prepared using MBs-BSA_FITC_ sandwiched between two blank hydrogels and filled into a 50-mm-long electrophoresis tube (outer diameter: 5 mm; inner diameter: 3 mm) for the EE. Subsequently, the filled channel was set in an electrophoresis device to construct the in-site EE model. The particle size of the MBs was larger than the pore size of the gel, and the fluorescent beads were physically immobilized in the gel while the excessive FITC was removed from the channel by electrophoresis. Before an EE run, the original fluorescence image of the target gel was captured using a CCD camera. After the 30-min EE (50 V, 6 mA, pH 7.4 PBS), the fluorescence image was also recorded by the CCD camera. The fluorescent images were converted to a grayscale intensity map. To simplify the calculation, a simple fluorescent image analysis method was developed. The side view of the grayscale intensity map is a two-dimensional plot of peaks. Each peak indicates a fluorescent spot at a given position along the length of the channel when the distribution density of the particles is low, and the peak value is the grayscale intensity of the fluorescent spot. The statistical peak numbers and values can be used to approximate fluorescent spots on the image. After image processing and calculations, 27.8% of the average grayscale intensity of the fluorescent spot was retained, comparing the average gray value of the bright spot before and after EE, and 97.6% of excessive FITC in the channel was cleared, obtained by calculating the decreased background fluorescence grayscale intensity after EE. The particle-to-background signal ratio (P/B ratio, PBr) increased from 1.08 to 12.2 after EE with an exposure time of 1.35 s. In addition, different exposure times were explored during the fluorescence detection. Increasing the exposure time from 1.35 to 2.35 s enhanced PBr from 12.2 to 15.5, which could effectively increase the signal-to-noise ratio. An appropriate increase in exposure time also allowed the detection of many weak fluorescent particles that were previously undetectable, indicating increased sensitivity of the fluorescence detection. The EE model has the following advantages: (i) increase in specificity by eluting FITC absorbed to the surface of beads; (ii) high efficiency in the removal of free FITC with more than 97% clearance; (iii) rapid decrease in noise in the mass hydrogel (within 30 min). This method can be used in beads/spots-based immunoassay in gel, immuno-electrophoresis, and fluorescent staining of protein/nucleic acid bands in gel electrophoresis.

微纳米颗粒具有高的比表面积适合表面修饰^[1,2]^,常用于核酸^[3]^、聚合物^[4]^、糖类^[5]^和蛋白质^[6]^等的灵敏检测分析。荧光成像可以探测免疫检测中荧光颗粒的位置^[7,8]^、平板凝胶中测定蛋白质或核酸条带^[9,10]^以及凝胶基质中染料的解吸附^[11⇓-13]^。免疫荧光检测通常需要对颗粒标记荧光探针,如异硫氰酸荧光素(FITC),用于指示生物^[14⇓-16]^或非生物分子^[17,18]^的结合或捕获。为此,各种荧光探针被开发用于生物目标物的标记^[19⇓-21]^。在标记过程中经常面临的一个问题是需要去除多余的染料探针,防止其产生的高背景荧光降低检测灵敏度^[22]^。目前常用的去除多余荧光染料的方法包括凝胶过滤^[23]^、透析^[24]^和电-膜萃取^[25]^。对于凝胶中的荧光颗粒,可通过连续洗涤进行纯化,但效率较低^[9⇓⇓⇓-13]^。过滤和透析对悬浮液中游离荧光探针的清除高效且低成本,但需要反复洗涤。尤其是基于流场驱动的反复洗涤难以高效地去除颗粒表面非特异性吸附的荧光探针^[23,24]^。电-膜萃取^[25]^结合液相微萃取和电泳的优点,从标记的人血清白蛋白(HSA)中高效地去除游离FITC,但是拦截蛋白质需要依赖膜孔径,且昂贵的滤板和滤膜等耗材导致纯化成本较高。上述方法或多或少需要面临耗时长、成本高、设备依赖性强等问题。

针对荧光颗粒的纯化,我们发展了一种电泳洗脱(electrophoretic elution, EE)模型,用于洗脱固定在凝胶基质中荧光标记颗粒上的多余荧光染料。用磁珠(MBs)、牛血清蛋白(BSA)和FITC分步合成荧光颗粒(MBs-BSA_FITC_),将MBs-BSA_FITC_溶液与凝胶混合后注入电泳通道(图1a)。将电泳管安放在电泳装置中进行EE,并用CCD相机成像(图1b)。通过记录EE前、后通道内的荧光图像定量分析游离FITC的清除率(图1c)。构建的EE模型可以模拟凝胶中基于颗粒的免疫测定、免疫电泳中抗原抗体结合或电泳中蛋白质荧光染色等场景。

**图 1:**
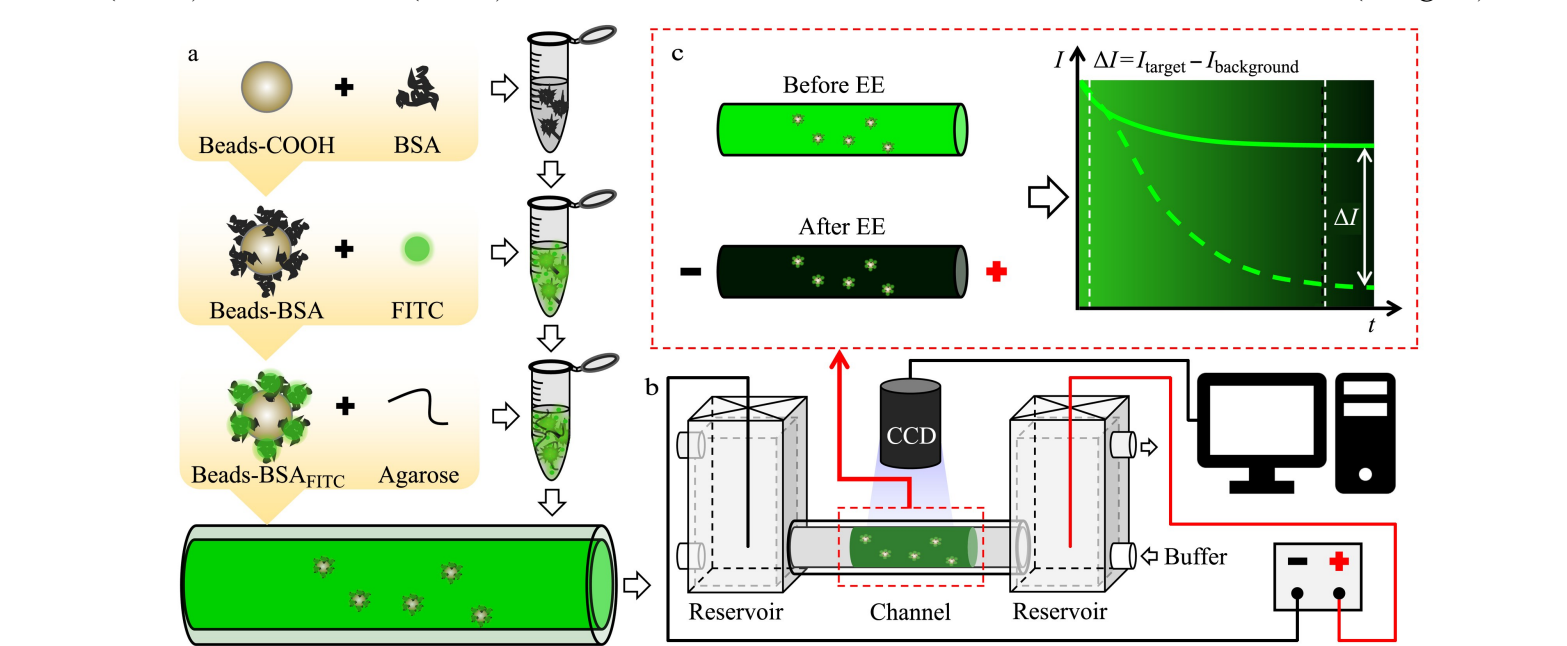
可荧光成像的电泳洗脱原理图

## 1 实验部分

### 1.1 仪器、试剂与材料

DYY-2C双稳定时电泳仪电源(北京六一生物科技有限公司),电泳仪(伯楷安生物科技有限公司),CCD相机(Olympus,日本)。

*N*-羟基琥珀酰亚胺(NHS)、1-(3-二甲基氨基丙基)-3-乙基碳二亚胺盐酸盐(EDC)、碳酸氢钠、碳酸钠、氯化钠购自麦克林试剂。BSA、低凝聚温度琼脂糖、2-(*N*-吗啡啉)乙磺酸(MES)、磷酸盐缓冲液粉剂购自Sigma-Aldrich。羧基磁珠(20 mg/mL,直径4.5 μm,蛋白质结合率(P/MB) 10~40 μg/mg)购自PuriMag Biotech。FITC购自罗恩试剂。离心管(EP管)购自Eppendorf。

配制40 mg/mL的BSA溶液用于MBs的蛋白偶联。每次活化羧基磁珠前,先用吗啉乙磺酸缓冲液(MEST,含0.01% Triton X-100, pH 6.0)分别制备50 mg/mL的EDC和NHS溶液。用7.56 g碳酸氢钠、1.06 g碳酸钠和7.36 g氯化钠制备1 L的交联反应液。低凝聚温度的琼脂糖(10 g/L)用于固定磁珠。混合步骤都在同一振荡仪(购自拓普森仪器有限公司)上完成。

### 1.2 蛋白偶联

取10 μL磁珠溶液到2 mL EP管,用200 μL MEST清洗3次,每次用磁铁将磁珠吸附于EP管壁并移除上清液。在EP管中分别加入100 μL 50 mg/mL EDC和NHS溶液以及1800 μL MEST。将EP管置于振荡仪上,在黑暗环境中振荡30 min。羧基被活化后,弃去上清液,并用200 μL MEST洗涤。加入400 μL BSA溶液和1600 μL MEST,振荡EP管10 h。孵育结束后,弃去上清液,即得到MBs-BSA。

### 1.3 FITC标记

向装有MBs-BSA的EP管中加入600 μL的FITC(1 mg/mL)和1400 μL交联反应液,将EP管置于4 ℃暗场环境孵育8 h。结束后利用磁铁去除上清液(此时管中剩余溶液5 μL),加入50 μL磷酸盐缓冲液(PBST,含0.01% Triton X-100, pH 7.4)复溶。将MBs-BSA_FITC_溶液与液态琼脂糖凝胶(10 g/L)混合(两者体积比为1:5),将150 μL混合凝胶与空白凝胶分段填入3 mm玻璃管。用CCD相机记录电泳通道内的荧光信号。

## 2 结果与讨论

图2a显示了通道内凝胶填充图像(图2a上)和EE前(图2a中)、后(图2a下)目标凝胶段的荧光图像。图2b是目标凝胶段荧光图像转化为灰度强度值的侧视图。EE前,大部分MBs-BSA_FITC_的荧光被背景荧光覆盖。EE后,游离FITC被洗脱降低了背景荧光强度,相当数量的荧光颗粒得以显现。图2c显示了荧光图像中可被裸眼观测的最亮和最暗颗粒的灰度强度,EE前的颗粒荧光灰度值较大但与背景区分度小。EE后,颗粒荧光灰度值减小,但与背景的区分度明显增大,微弱荧光颗粒更容易被检测到。如图2d所示,颗粒和背景荧光信号灰度强度值分别从154.9和142.9降低到43.1和3.5。给定游离FITC的清除率为EE前、后通道内背景荧光灰度强度减少的百分比,颗粒荧光保留率为EE前、后的颗粒荧光灰度强度比值^[25]^。据此,颗粒荧光保留了27.8% (43.1/154.9),而通道内背景荧光减少了97.6% ((142.9-3.5)/142.9)。EE后,颗粒与背景的信号强度比值(PBr)从1.08 (PBr=154.9/142.9)增加到12.2 (PBr=43.1/3.5)。

**图 2:**
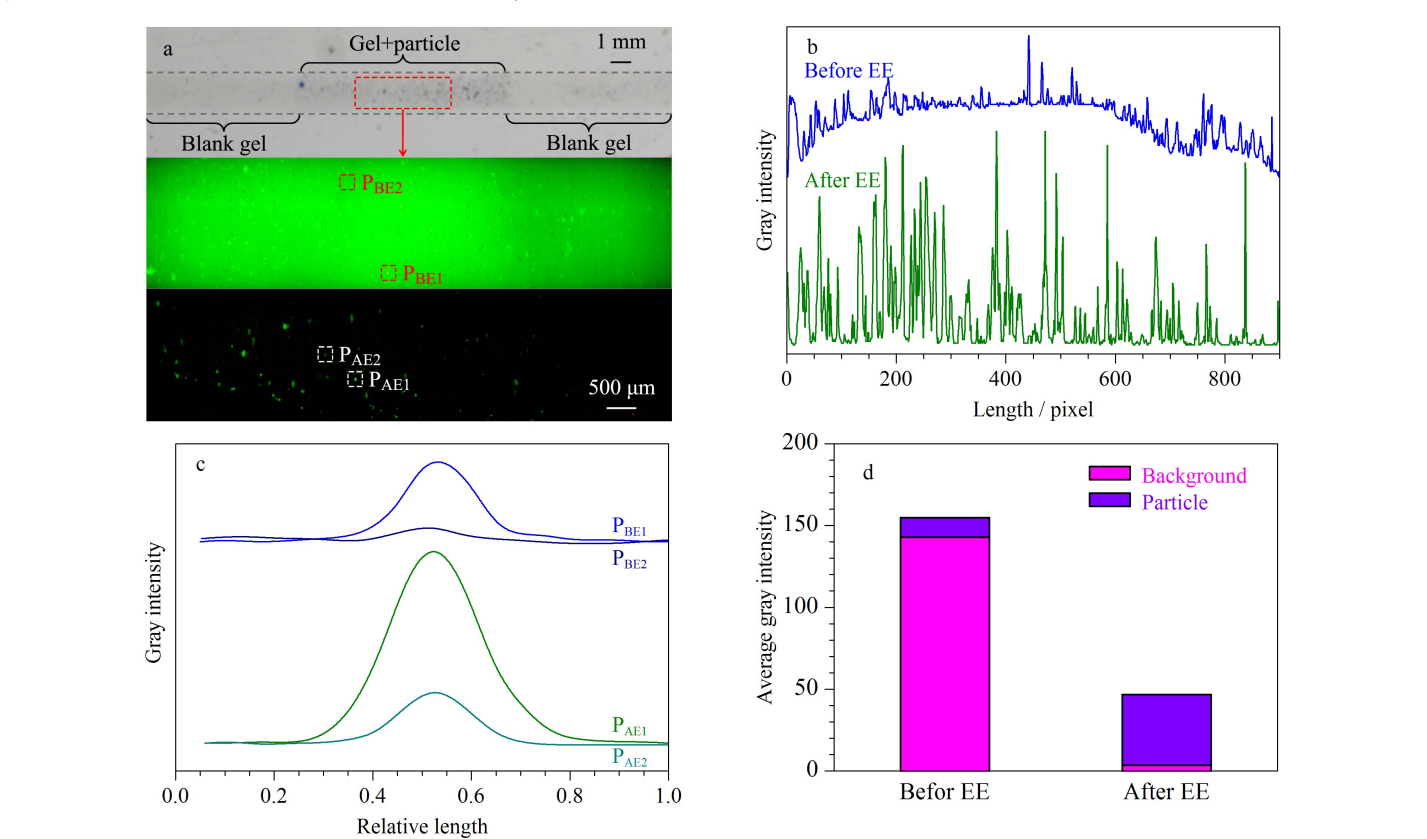
目标凝胶段的荧光图像以及相关灰度数据

激发光(488 nm)照射下,FITC发出的荧光强度可简单表示为浓度的函数^[26]^(*I*_FITC_=*K*·*c*_FITC_,给定检测系统后可认为*K*为常数)。考虑曝光时间(Δ*t*)影响^[27]^,图像荧光强度可表示为*I*_im_=*I*_FITC_ · Δ*t*=*K*·*c*_FITC_ ·Δ*t*。

根据上述关系,估算EE前、后凝胶中FITC的含量。标记时加入的FITC浓度为 $c_{FITC}^{Label}$ =1 mg/mL· 600 μL/2 mL/389=771.21 μmol/L,去除上清液(剩余的溶液体积为5 μL)并溶解在50 μL PBST溶液中,对应的FITC浓度为 $c_{FITC}^{PBST}$ =771.21 μmol/L·5 μL/55 μL=70.11 μmol/L,与凝胶溶液1:5混合,对应浓度是 $c_{FITC}^{Gel}$ =70.11 μmol/L·55 μL/330 μL=11.69 μmol/L。系统相关参数为*K=* $I_{im}^{B-BE}$ / $c_{FITC}^{Gel}$ /Δ*t*=142.9/11.69 μmol/L/1.35 s=9.05 L/(μmol·s),上标B-BE表示电泳洗脱前的背景荧光。电泳洗脱后通道中的FITC残留量估计有 $c_{FITC}^{Res}$ = $I_{im}^{B-AE}$ /*K*/Δ*t*=3.5/9.05 L/(μmol·s)/1.35 s=0.29 μmol/L,上标B-AE表示电泳洗脱后的背景荧光。

磁珠上的荧光清除率为72.2% ((154.9-43.1)/154.9),洗脱后颗粒荧光强度明显下降,可能原因是颗粒荧光包含3个部分:MBs-BSA_FITC_上有效标记的FITC,吸附在颗粒表面的游离FITC,以及颗粒间微体积溶液中的游离FITC。其中,吸附在颗粒表面^[28]^和微体积溶液中的游离FITC被电泳洗脱,造成颗粒荧光和背景荧光强度下降。结果表明,电泳洗脱能洗去大部分噪声信号,同时保留有效标记的FITC荧光信号,提高了荧光信号的选择性和检测灵敏度。

相同荧光强度的颗粒均匀分布在通道中可以直观地反映出荧光标记物在颗粒上的覆盖情况^[29]^。然而磁珠颗粒尺寸分布存在一定范围,FITC在磁珠表面的结合不均匀,且MBs-BSA_FITC_的制备和凝胶灌注过程中存在颗粒的局部聚集。这些现象的出现导致荧光图像难以用单一阈值来划分提取荧光颗粒。考虑到通道中颗粒分布密度较低,采用灰度强度侧视图进行相关统计(见附图S1~S3, https://www.chrom-China.com/)。图像中亮点数量和灰度强度值能近似表示FITC含量。

荧光图像是CCD相机在给定曝光时间内捕获荧光信号的集合。图3a是电泳洗脱后的目标凝胶段在不同曝光时间下的荧光图像。曝光时间越长,图像荧光强度就越大,可观察到的荧光颗粒数量增多。计算灰度强度侧视图的峰数量、总峰面积和平均峰间隔。长曝光时间下,背景荧光也会增加,因此计算中进行基线校正^[30]^(见附图S4)。图3b表明,基线校正会导致灰度峰数目的损失,这主要是对背景荧光的过滤所引起的。图3c中,校正前总峰面积是图像中达到强度阈值区域的面积,校正后的结果则反映了荧光颗粒的面积。图3d表明颗粒随机散布,基线校正处理对荧光颗粒分布统计的均匀性不会造成影响。

**图 3:**
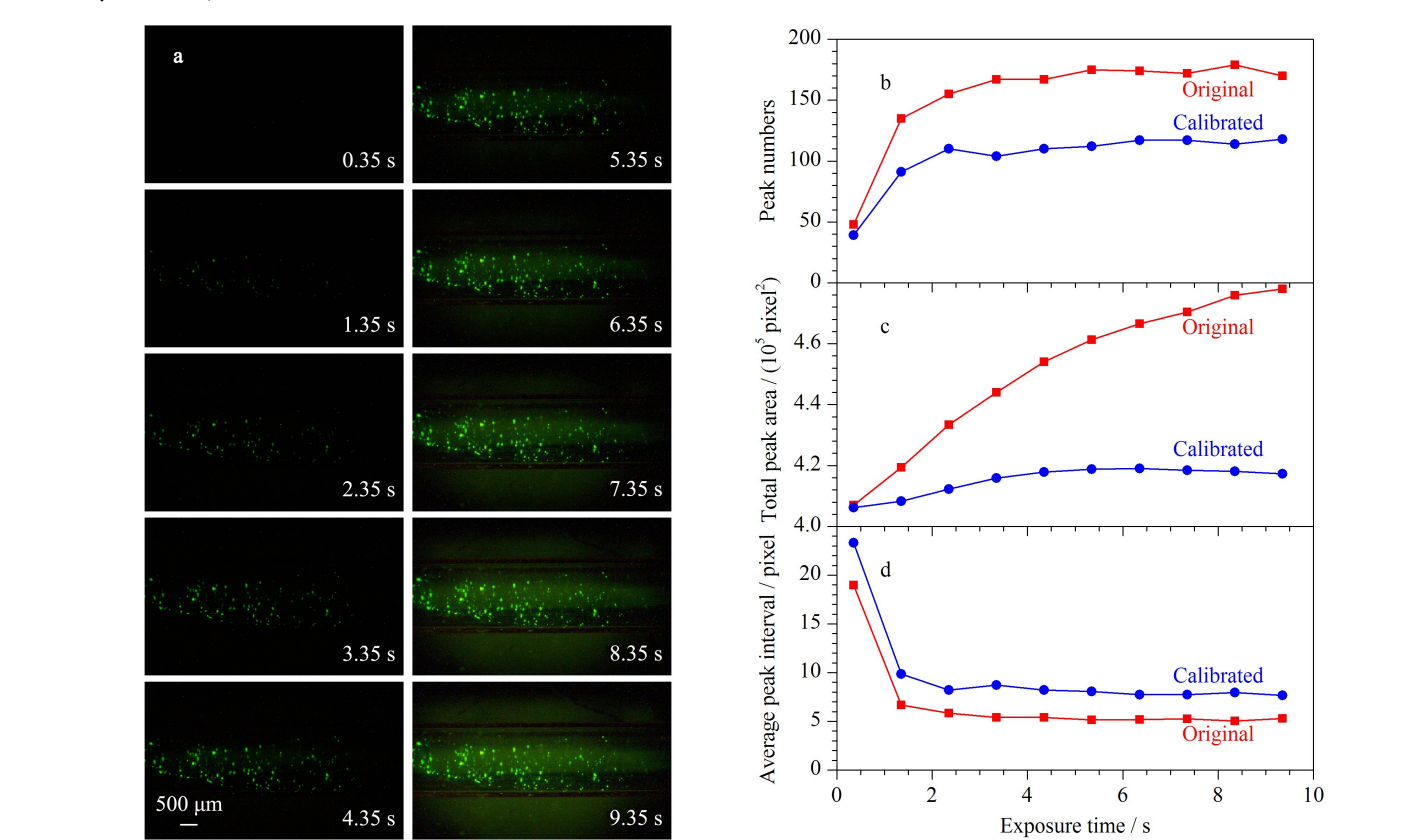
不同曝光时间下的电泳洗脱后荧光图像以及相关灰度数据

增加曝光时间能提高荧光检测灵敏度,因为颗粒和背景荧光的累积存在差异,PBr有所提高。但过分延长曝光时间,通道中残留的FITC会让背景荧光强度同样增加,PBr趋于稳定。因此适当延长曝光时间(Δ*t*=2.35 s)可以提高荧光颗粒的检测灵敏度,得到较高的PBr(PBr=58.72/3.78=15.52) (见附图S5)。

## 3 结论

本文提出的电泳洗脱模型利用凝胶固定颗粒,通过电泳洗脱多余荧光染料,发展的MBs-BSA_FITC_荧光图像检测方法可以初步估算多余FITC的去除率。电泳洗脱去除吸附在颗粒表面的FITC和溶液中游离的FITC,提高了荧光颗粒检测方法的特异性和灵敏度。适当增加曝光时间可以提高颗粒和背景信号比值。该方法具有以下优点:(1)提高了荧光颗粒检测的特异性;(2)快速降低了凝胶中的背景噪声,提高了检测灵敏度;(3)清除游离FITC的效率高,超过97%。该模型可用于清除荧光标记颗粒、免疫电泳中蛋白质/核酸标记以及凝胶电泳中荧光染色等各种不同场景中的多余荧光染料。

## References

[1] Calmo R, Chiadò A, Fiorilli S, et al. ACS Appl Bio Mater, 2020, 3(9): 5787 10.1021/acsabm.0c00533 35021809

[2] Markwalter C F, Kantor A G, Moore C P, et al. Chem Rev, 2019, 119(2): 1456 30511833 10.1021/acs.chemrev.8b00136 PMC6348445

[3] Diebbi K, Shi B, Weng T, et al. ACS Omega, 2022, 7(2): 2224 35071911 10.1021/acsomega.1c05775 PMC8771974

[4] Andel E V, Bus I D, Tijhaar M E J, et al. ACS Appl Mater Interfaces, 2017, 9(44): 38211 29064669 10.1021/acsami.7b09725 PMC5682608

[5] Gao W J, Bai Y, Liu H W. Chinese Journal of Chromatography, 2021, 39(9): 981 34486837 10.3724/SP.J.1123.2021.08012 PMC9404082

[6] Zhou X X, Zhang Y J, Kang K, et al. Anal Chem, 2022, 94(11): 4650 35254814 10.1021/acs.analchem.1c04587

[7] Silva-Santos A R, Oliveira-Silva R, Rosa S S, et al. ACS Appl Nano Mater, 2021, 4(12): 14169

[8] You P Y, Li F C, Liu M H, et al. ACS Appl Mater Interfaces, 2019, 11(10): 9841 30784256 10.1021/acsami.9b00204

[9] Pruthi R K, Daniels T M, Heit J A, et al. Thrombosis Res, 2010, 126(10): 543 10.1016/j.thromres.2010.09.015 20889192

[10] Brüchert S, Joest E F, Gatterdam K, et al. Commun Biol, 2020, 3: 138 32198384 10.1038/s42003-020-0852-1 PMC7083852

[11] Ludes M D, Anthony S R, Wirth M J. Anal Chem, 2003, 75(13): 3073 12964753 10.1021/ac0264170

[12] Feng J J, Ji X P, Li C Y, et al. Chinese Journal of Chromatography, 2021, 39(8): 781 34212580 10.3724/SP.J.1123.2021.02030 PMC9404022

[13] Zhu Q Z, Trau D. Biosens Bioelectrons, 2015, 66(15): 370 10.1016/j.bios.2014.10.083 25463645

[14] Shaner N C, Steinbach P A, Tsien R Y. Nat Methods, 2005, 2(12): 905 16299475 10.1038/nmeth819

[15] Zhang M, Le H N, Ye B C. ACS Appl Mater Interfaces, 2013, 5(17): 8278 23968374 10.1021/am402429n

[16] Wang Z G, Liu S L, Pang D W. Acc Chem Res, 2021, 54(14): 2991 34180662 10.1021/acs.accounts.1c00276

[17] Zhu Y P, Ma T Y, Ren T Z, et al. ACS Appl Mater Interfaces, 2014, 6(18): 16344 25163834 10.1021/am504554h

[18] Zhao Y J, Bernitzky R H M, Kory M J, et al. J Am Chem Soc, 2016, 138(28): 8976 27347597 10.1021/jacs.6b05456

[19] Fujii T, Shindo Y, Hotta K, et al. J Am Chem Soc, 2014, 136(6): 2374 24447167 10.1021/ja410031n

[20] Ding P, Wang H, Song B, et al. Anal Chem, 2017, 89 (15): 7861 28621521 10.1021/acs.analchem.6b04427

[21] Makino K, Susaki E A, Endo M, et al. J Am Chem Soc, 2022, 144(4): 1572 35048690 10.1021/jacs.1c09844

[22] Komatsu T, Kikuchi K, Takakusa H, et al. J Am Chem Soc, 2006, 128(50): 15946 17165702 10.1021/ja0657307

[23] Chen D D, Yuan Y, Yu J B, et al. Anal Chem, 2018, 90 (9): 5569 29569904 10.1021/acs.analchem.8b00095

[24] Mikheikin A, Olsen A, Picco L, et al. Anal Chem, 2016, 88(5): 2527 26878668 10.1021/acs.analchem.5b04023

[25] Restan M S, Pedersen M E, Jensen H, et al. Anal Chem, 2019, 91(10): 6702 31038306 10.1021/acs.analchem.9b00730

[26] Liebsch G, Klimant I, Krause C, et al. Anal Chem, 2001, 73(17): 4354 11569831 10.1021/ac0100852

[27] Sandén T, Persson G, Thyberg P, et al. Anal Chem, 2007, 79(9): 3330 17385841 10.1021/ac0622680

[28] Kästner C, Böhmert L, Braeuning A, et al. Langmuir, 2018, 34(24): 7153 29792806 10.1021/acs.langmuir.8b01305

[29] Wang Z, Wang X, Liu S, et al. Anal Chem, 2010, 82(23): 9901 21062024 10.1021/ac102416f

[30] Ning X, Selesnick I W, Duval L. Chemometr Intell Lab, 2014, 139: 156

